# A Manual of Hypodermatic Medication

**Published:** 1892-02

**Authors:** 


A MANUAL OF HYPODERMATIC MEDICATION.—The treatment of Diseases by the Hypodermic or Subcutaneous Method. By Roberts Bartholow, A. M., M. D., LL. D., Emeritus Professor of Materia Medica, General Therapeutics and Hygiène in the Jefferson Medical College of Philadelphia; Author of A Treatise on the Practice of Medicine; A Treatise on Materia Medica and Therapeutics; Author of a Manual of Medical Electricity, etc., etc. Fifth Edition. Revised and Enlarged. J. B. Lippincott & Co., Philadelphia, 1891. Price, $3.00, in cloth.

This is a neat volume of 540 pages, having been revised and enlarged four times. It is written in plain and neat style, giving the author's views and experiments of medication by subcutaneous injection, as well as the views of other men of experience in this line of treatment.

The many late experiments in the treatment of tuberculosis and lupus have revived this mode of treatment.

Valuable information is here given in regard to the dosage and menstruum of the drug to be used.

The name of the author is sufficient to assure us of its merit.

After a careful examination of its pages, I can truthfully say that it is a work that every physician ought to possess.

It is a work that is creditable to the author. J. M. C.

